# Involvement of Multiple Transporters-mediated Transports in Mizoribine and Methotrexate Pharmacokinetics

**DOI:** 10.3390/ph5080802

**Published:** 2012-08-10

**Authors:** Teruo Murakami, Nobuhiro Mori

**Affiliations:** Laboratory of Biopharmaceutics and Pharmacokinetics, Faculty of Pharmaceutical Sciences, Hiroshima International University, 5-1-1 Hiro-koshingai, Kure, Hiroshima 737-0112, Japan; Email: n-mori@ps.hirokoku-u.ac.jp

**Keywords:** oral bioavailability, interindividual variability, intraindividual variability, transporter-mediated transport, polymophisms, therapeutic drug monitoring

## Abstract

Mizoribine is administered orally and excreted into urine without being metabolized. Many research groups have reported a linear relationship between the dose and peak serum concentration, between the dose and AUC, and between AUC and cumulative urinary excretion of mizoribine. In contrast, a significant interindividual variability, with a small intraindividual variability, in oral bioavailability of mizoribine is also reported. The interindividual variability is mostly considered to be due to the polymophisms of transporter genes. Methotrexate (MTX) is administered orally and/or by parenteral routes, depending on the dose. Metabolic enzymes and multiple transporters are involved in the pharmacokinetics of MTX. The oral bioavailability of MTX exhibits a marked interindividual variability and saturation with increase in the dose of MTX, with a small intraindividual variability, where the contribution of gene polymophisms of transporters and enzymes is suggested. Therapeutic drug monitoring of both mizoribine and MTX is expected to improve their clinical efficacy in the treatment of rheumatoid arthritis.

## 1. Introduction

Mizoribine and methotrexate (MTX) are immunosuppressants and used in the treatment of rheumatoid arthritis (RA). Recently, it was reported that the combination therapy with mizoribine and MTX was effective and relatively safe to control the symptoms of RA in patients with an insufficient clinical response to MTX alone [1,2]. Mizoribine and MTX are administered orally, though MTX is also administered by parenteral routes.These drugs are hydrophilic and cannot penetrate lipoidal biomembranes by passive diffusion. Their membrane transports are mediated by multiple solute carrier (SLC) influx transporters. ATP binding cassette (ABC) efflux transporters are also involved in the membrane transport of MTX. In pharmacotherapy, both drugs are known to show a significant interindividual variability in the oral bioavailability, though the intraindividual variability is relatively small. In this chapter, the characteristic pharmacokinetics of mizoribine and MTX are reviewed from the viewpoints of transporter-mediated transports and polymophisms in genes involved in transporters.

## 2. Introduction for Mizoribine

**Figure 1:**
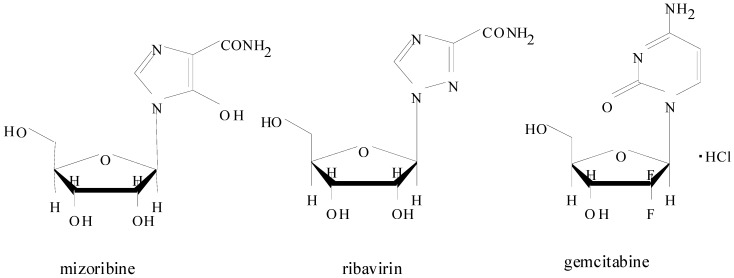
Chemical structures of mizoribine (a CNT1, CNT2 substrate), ribavirin (CNT2 substrate) and gemcitabine (CNT1 substrate).

Mizoribine (Bredinin^®^, 4-carbamoyl-1-β-D-ribofuranosylimidazolium-5-olate), with pka values of 1.4 for the 5-hydroxy group (O^−^) and 6.75 for the 3-imino group (NH^+^), is a nucleoside of the imidazole class (Figure 1). In Figure 1, the chemical structures of ribavirin and gemcitabine are also shown.

Mizoribine was originally isolated in Japan in 1971 from the culture filtrate of *Eupenicillium brefeldianum* as a substance having weak antimicrobial activity against *Candida albicance* [3]. Thereafter, mizoribine-5′-monophosphate, a phosphorylated metabolite in the cell, was found to show cytotoxicity by inhibiting key enzymes such as inosine 5′-monophosphate (IMP) dehydrogenase and guanosine-monophosphate (GMP) synthetase. Consequently, guanine ribonucleotide in the cells is depleted, leading to inhibition of DNA synthesis and reduction of cell proliferation. Mizoribine has been on the market in Japan since 1984, and is used in clinical practice as a basic immunosuppressant in kidney transplantation and in the treatment of autoimmune diseases such as lupus nephritis, nephritic syndrome, and rheumatoid arthritis (RA) [3,4,5,6,7,8]. Mizoribine was also found to inhibit malaria parasite and hepatitis C virus (HCV) RNA replication, and is considered to be a potential use as a new antimalarial chemotherapy and anti-HCV reagent in combination with interferon-α, respectively [9,10]. Mizoribine needs some specific transport system, or nucleoside transporters, to penetrate lipoidal biomembranes, since mizoribine is a water soluble hydrophilic compound with a very low lipophilicity log P is −2.87 calculated by Crippen’s fragmentation method) [11]. Nucleoside transporters are divided into two categories: Na^+^-independent equilibrative nucleoside transporter (ENT) that is known as a facilitated diffusion system and Na^+^-dependent concentrative nucleoside transporter (CNT) that is known as an active transport system [12]. ENT family has four members (ENT1, ENT2, ENT3, ENT4) having 11 transmembrane domains and a glycosylated extracellular loop, and these proteins are located in the basolateral membrane of absorptive epithelia [13]. CNT family contains three members (CNT1, CNT2, CNT3) having 13 transmembrane domains and a glycosylated C-terminus, and these proteins are located in the apical membrane of absorptive epithelia. This indicates that substrate compounds are actively transported into cells by CNTs via apical membrane and then effluxed passively into blood circulation from cells by using facilitated transporter ENTs via basolateral membrane of absorptive epithelia. CNT1 and CNT2 are expressed in a proximal-to-distal gradient along the rat intestine, whereas the expression level of CNT3 is quite low in rats [14,15]. CNT1 transports uridine, thymidine, cytidine (pyrimidine nucleosides) and adenosine (a purine nucleoside), and CNT2 transports guanosine, adenosine (both purine nucleosides) and uridine (a pyrimidine nucleoside). CNT3 transports both purine and pyrimidine nucleosides [12,13,16,17]. Mizoribine is transported by CNT1 and CNT2 [18]. Gemcitabine, a pyrimidine analogue, is transported by CNT1 and CNT3, in addition to ENT1 and ENT2, but not by CNT2 [14,19,20], and ribavirin is transported by CNT2 [21].

There is a positive relationship between the serum level of mizoribine and its clinical efficacy without causing severe adverse effects [22,23,24]. This indicates that pulse therapy or high-dose of mizoribine is applicable in clinical use. For example, Kawasaki *et al.* [24] reported that oral mizoribine pulse therapy consisting of a single dose two days a week may be effective and safe in some frequently relapsing nephritic syndrome patients. The membrane transport of mizoribine, a very water soluble hydrophilic compound, is mediated by nucleoside transporters, and therefore the extent of absorption of mizoribine is affected by various factors such as the dose of mizoribine (saturation), interindividual variability in functional expression of intestinal transporters and luminal proton concentration, and polymorphism of transporters. Mizoribine distributed into cells by transporter-mediated transport is metabolized to mizoribine-5′-phosphate, an active metabolite, by adenosine kinase in cells, and the mizoribine-5′-phosphate selectively inhibits inosine monophosphate (IMP) dehydrogenase that catalyses the conversion of IMP to xanthiosine monophosphate (XMP) [25,26]. Accordingly, mizoribine causes the accumulation of IMP and the reduction of XMP, resulted in the inhibition of cell growth. Mizoribine is excreted into urine as an intact form depending on the glomerular filtration rate (GFR) of each patient. Thus the plasma disposition, or total body clearance or AUC, of mizoribine is largely dependent on patient’s renal function. In the following section, the pharmacokinetics of mizoribine are reviewed.

### 2.1. Pharmacokinetics of Mizoribine in Human Subjects

Stypinski *et al.* [27] reported the pharmacokinetics of higher-dose mizoribine in healthy male volunteers. In that study, orally administered mizoribine reached peak serum concentrations within 2–3 h and was eliminated mostly via the kidney with a 3 h half-life as follows: urinary excretion at a dose of 3 mg/kg was 65 ± 22% of dose, at 6 mg/kg 104 ± 27%, at 9 mg/kg 79 ± 35% and at 12 mg/kg 78 ± 15%. Approximately a 2-fold difference was observed in the values of the individual maximum serum concentration (C_max_) and area under the concentration-time curve from time zero to infinity (AUC) among patients. Takada *et al.* [28] studied the pharmacokinetics of mizoribine in renal transplant patients and reported a linear relationship between the peak serum levels and the oral dose of mizoribine in a dosing range of 0.85–4.46 mg/kg. In that study, peak serum mizoribine concentration was observed 2.4 h after oral administration and the calculated mean peak serum level was 0.852 μg/mL/mg dose/kg body weight. The elimination rate constant of mizoribine was well correlated with the endogenous creatinine clearance, suggesting the necessity of dosage adjustment of mizoribine based on the renal function of the renal transplant patient. Warabi *et al.* [29] examined the pharmacokinetics of mizoribine after consequtive administration at a dose of 50 mg in RA patients and reported that the urinary excretion of mizoribine was 52.6 ± 19.7% of dose on the first day and 57.2 ± 25.2% after repeated administration. Similar results also have been obtained at a dose of 100 mg in RA patients, indicating that there is no accumulation of mizoribine in the body even after multiple repeated administrations of mizoribine. In the study, a linear relationship was observed between the dose and peak serum concentration of mizoribine, between the dose and AUC, and between AUC and cumulative urinary excretion of mizoribine. Ihara *et al.* [30] reported that the cumulative urinary excretion of mizoribine, or oral bioavailability, in kidney transplant recipients was variable and ranged from 12% to 81% of the dose (in total 41 ± 22% of dose). In contrast, the coefficient of variation (CV%) of bioavailability of mizoribine in intraindividual measurements (mean 14.4%) was much smaller than that in interindividual measurements (45.5%). In the study, the volume of distribution (Vd) ranged from 0.18 to 0.57 (0.39 ± 0.13) L/kg. A linear relationship was observed between the age of patients and bioavailability (F) of mizoribine (n = 14, F = −0.01 x age + 0.83, r = 0.538, *p* < 0.05) and between creatinine clearance (CLcr) and total body clearance (CLB) of mizoribine (n = 24, CLB = 1.09 x CLcr − 4.22, *p* < 0.0001). Tanaka *et al.* [20] reported that the peak level of mizoribine in the serum was significantly correlated with the single dose of mizoribine (r = 0.509, *p* = 0.0371), and that a significant inverse correlation (r = −0.596, *p* = 0.0116) was observed between the peak serum levels of mizoribine and the serum anti-dsDNA antibody titers in patients with clinically stable lupus nephritis. Abe *et al.* [31] examined the pharmacokinetics of mizoribine in child-onset glomerulonephritis by administering mizoribine orally at 60–300 mg/day (3.0–8.4 mg/kg/day) divided in one or two daily doses. At a dose of 3.36 ± 1.91 mg/kg of mizoribine, the time to peak serum concentration (T_max_) was 2.94 ± 0.82 h, peak serum level (C_max_) 1.59 ± 1.16 μg/mL, half-life t_1/2_ 1.96 ± 0.92 h, AUC 9.36 ± 6.58 μg·h/mL, volume of distribution at a steady state (Vdss) 2.03 ± 0.80 L/kg, and rate of urinary excretion was 49.1 ± 18.7% of dose.

### 2.2. Effect of Polymorphisms on Mizoribine Bioavailability

Naito *et al.* [32] analysed the disposition of mizoribine pharmacokinetically based on polymorphism of CNT1 gene in kidney transplant recipients. The median bioavailability of mizoribine was 44.8%, with interindividual variability (interquartile range, 37.8%–61.5%). The correlation coefficient between creatinine clearance and renal clearance of mizoribine was 0.65. The median mizoribine bioavailability was 42.0% in CNT1 565GA, 41.4% in 565AA and 62.4% in 565GG. The CNT1 G565A allele frequency was 51.5% and was suggested to contribute to interindividual differences in plasma disposition of mizoribine. Fukao *et al.* [33] investigated the genetic factors responsible for the interindividual variability in the bioavailability of mizoribine, in which the oral bioavailability of mizoribine in the 30 subjects ranged from 60.3% to 99.4% when mizoribine was administered at a dose of 150 mg. The mean oral bioavailabilities of mizoribine in subjects with the CNT1 565AA allele (five subjects), CNT1 565GA (18 subjects), and CNT1 565GG (seven subjects) were 75.4%, 86.7%, and 90.1% of dose, respectively, and the value in subjects with the CNT1 565AA allele was significantly lower than that in subjects with CNT1 565GG allele. In the study, the effect of breast cancer resistant protein (BCRP) and multidrug resistance-associated protein 4 (MRP4) polymorphisms on the bioavailability of mizoribine in healthy subjects was not detected.

### 2.3. Pharmacokinetics of Mizoribine in Animal Studies

Murase *et al.* [34] examined the pharmacokinetics of mizoribine in rats using ^14^C-labeled compound. After oral administration (9 mg/kg), the radioactivity in blood reached a maximum at 1.5 h, decreased rapidly thereafter, and almost disappeared at 24 h. Within 24 h after dosing, 85% of the dose was excreted in the urine and 9.7% in the feces, and less than 1% in the bile, and within 96 h after dosing, 86.4% of the dose was excreted in the urine and 11.8% in the feces. Also, it was found that more than 99% of the radioactivity in plasma at 1 h after dosing was due to the unchanged mizoribine, and mizoribine was found to be excreted mainly unchanged in the urine. Uchida *et al.* [35] reported that there was no accumulation of mizoribine in blood even after repeated oral administrations for 36 consecutive months (10 mg/kg once a day, 6 days a week, with a day-off on the 7th of each week) in beagle dogs. To *et al.* [36] examined the chronopharmacological profiles of mizoribine, since other antimetabolites such as MTX and 6-mercaptopurine have been shown to have circadian variations in their toxicities in rats. The AUC value of mizoribine administered orally at 8:00 h was twice as high as that administered at 20:00 h, and the group treated at 8:00 h showed severe myelosuppression compared with the group treated at 20:00 h.

Okada *et al.* [21] reported that the absorption rate of mizoribine in the most proximal segment of intestinal loop was the highest, and the intestinal absorption of mizoribine was significantly decreased in the presence of inosine (a CNT2 substrate), or by the pretreatment with inosine or inosinic acid in rats, suggesting the contribution of CNT2 (or N1 transporter) in the intestinal absorption of mizoribine. Mori *et al.* [18] examined the characteristics of intestinal absorption of mizoribine from the viewpoints of the contribution of CNT1 and CNT2 in rats. The urinary excretion percentage of unchanged mizoribine was a dose dependent: 53.1% at 5 mg/kg and 24% at 20 mg/kg. When mizoribine was administered into the intestinal loops, the disappearance rate of mizoribine from the loop was comparable between 1 and 5 mg/kg, but significantly lower at 25 mg/kg. Coadministration of adenosine (a substrate of both CNT1 and CNT2), thymidine (a CNT1 substrate), inosine (a CNT2 substrate), gemcitabine (a CNT1 substrate) and ribavirin (a CNT2 substrate) significantly suppressed the mizoribine intestinal absorption. This indicated that the intestinal absorption of mizoribine is mediated by CNT1 and CNT2. The suppression of ribavirin uptake by LS180 cells in the presence of mizoribine (5 mM) is also reported by Takaai *et al.* [37]. In the study by Mori *et al.* [18], bile and bile salts such as sodium cholate and sodium glycocholate (10 mM) were also found to cause interaction with mizoribine. The effects of bile or bile salts on the intestinal absorption of mizoribine were further studied in cholestatic rats [38] and in lipopolysaccharide (LPS)-treated rats [39], in which cholestatic states were induced by the treatment with either carbontetrachloride (CCl_4_) or α-naphthyl- isothiocyanate (ANIT). In these CCl_4_-, ANIT-, and LPS-treated rats, the oral bioavailability of mizoribine was increased a little, but significantly, possibly due to its less amounts of bile in the intestinal lumen. The intestinal absorption site of mizoribine was studied in rats by examining the effects of altered the gastric emptying rates (GERs) on intestinal absorption [38]. The altered GERs exerted no significant effects on peak plasma level (C_max_), AUC_0-6_, and urinary excretion percentage of mizoribine administered orally, though the time to reach C_max_ (T_max_) of mizoribine was altered when GERs were altered. These results suggested that mizoribine is absorbed efficiently to the same extents from the whole small intestine in rats.

### 2.4. Summary for Mizoribine Pharmacokinetics

It is generally accepted that mizoribine is rapidly absorbed from the gastrointestinal tract and excreted into urine as an unchanged form at a percentage of 65%–100% of dose in healthy human subjects [27]. The disposition of mizoribine from blood circulation is highly dependent on renal function [28,31,40]. In contrast, however, a marked interindividual variability in oral bioavailability of mizoribine is also reported, in which the oral bioavailability of mizoribine ranged from 12% to 81% of the dose (in total 41 ± 22% of dose) in kidney transplant recipients [30]. On one hand, the intraindividual variability in oral bioavailability of mizoribine reported by various investigaters is relatively small. These phenomena of mizoribine pharmacokinetics may suggest that the marked interindividual variability in oral bioavailability of mizoribine comes from the variation of functional expression of transporters in each patients and/or variation of individual disease states. Such marked interindividual variability in oral bioavailability of mizoribine was partly explained by polymorphism of CNT1 gene [32,33]. However, it is also considered that the polymorphism of CNT1 gene alone could not explain such a marked interindividual variability in mizoribine bioavailability, though the effect of CNT1 polymorphism was potential. Taken together, at present, therapeutic drug monitoring (TDM) of mizoribine will be important to overcome such a marked interindividual variability in clinical practice with mizoribine. Further study is needed to clarify the mechanism of interindividual variability in mizoribine oral bioavailability under disease states.

## 3. Introduction for Methotrexate

**Figure 2:**
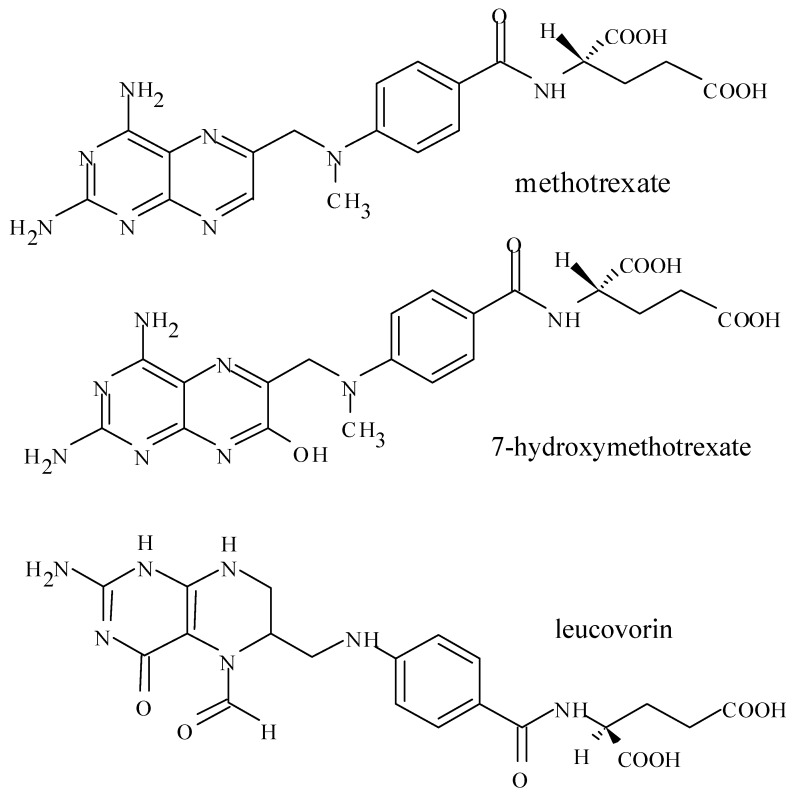
Chemical structures of methotrexate, 7-hydroxymethotrexate and leucovorin.

Methotrexate (MTX, N-[4-[[(2,4-diamino-6-pteridinyl)methyl]methylamino]-L-glutamic acid), having a similar chemical structure to folic acid, is an antifolate drug. MTX containes one glutamic acid and therefore has two carboxylate groups with pKa 4.8 and 5.5 in its chemical structure (Figure 2). At a physiological pH, MTX is ionized and hydrophilic. MTX poorly penetrates lipoidal biomembranes by passive diffusion, but penetrates by carrier-mediated transport system. At a low pH, MTX is in unionized form and sparingly soluble.

In the urinary excretion process, MTX may be precipitated in the urine and cause renal damage, if the pH of urine is low [41]. Patients receiving high-dose MTX must receive hydration fluids and sodium bicarbonate orally and/or intravenously to alkalize pH of urine. In pharmacotherapy with high-dose MTX, MTX is administered by the parenteral routes. When administered intravenously, the central volume of distribution (V1) of MTX is relatively low, 0.2 L/kg, which is corresponding to the water volume of plasma and intercellular space of tissues. In addition, the volume of distribution based on area under the concentration-time curve (Vd AUC), estimated from the dose of drug (X_0_), elimination rate constant at a β phase (β) and AUC value using the following equation: Vd, AUC = X_0_/β·AUC, is 0.7 L/kg. This value roughly corresponds to the total water volume in human body. At a physiological pH, MTX exists in ionized form, and therefore MTX distributes various tissues by using specific transport system for folic acid, and dissolved in water fraction. The protein binding of MTX is about 50% and MTX is primarily excreted into the urine (more than 80% as an intact form). The total clearance of MTX is approximately 1.6-fold of creatinine clearance, indicating the contribution of urinary secretion to some extent in addition to the glomerular filtration. The elimination half lives of MTX after intravenous administration are 3 h and 10 h for the α and β phases, respectively [41].

A part of MTX is metabolized to 7-hydroxymethotrexate (7-OH-MTX) mainly by aldehyde oxidase [42], 2,4-diamino-N10-methylpteroic acid (DAMPA) by intestinal bacteria [43] and polyglutamate derivatives (MTX-PGs) by folylpoly-γ-glutamate synthetase in cells [44,45,46,47]. MTX-PGs are hydrolyzed by γ–glutamyl hydrolase in cells of humans and various animal species [48,49]. Seideman *et al.* [50] determined the plasma concentrations of MTX and 7-OH-MTX over 7 days and their urinary excretion over 24 h after intravenous, oral and intramuscular administration of 15 mg MTX in RA patients.

The AUC values of MTX did not differ between intra-muscular and oral administrations, indicating the similar bioavailability after these routes of administration. The AUC of 7-OH-MTX was also similar after these two routes of administration. Over 80% of MTX (96.7% after intravenous, 80.5% after oral, and 88.5% after intramuscular administration) was excreted into urine as an intact drug and about 3% was excreted as 7-OH-MTX during 24 h after administration. Uchiyama *et al.* [51] developed a simple and sensitive HPLC method and measured the plasma concentrations of MTX, 7-OH-MTX, and DAMPA in a patient receiving a high-dose MTX infusion (3,000 mg/m^2^ for 2 h). The plasma level of DAMPA 24 h, 48 h, and 96 h after administration was less than the detection limit (3.4 ng/mL), though higher concentrations of 7-OH-MTX were observed.

In cells, MTX is metabolized to MTX-PG by folypolyglutamate synthetase, and MTX-PG binds and inactivates the dihydrofolate reductase (DHFR), thymidylate synthethase (TYMS) and aminoimidazole carboxamide ribonucleotide transformylase (ATIC) [49]. DHFR is an enzyme that catalyses the conversion of dihydrofolate (DHF) to the active tetrahydrofolate (THF), which is important in the production of 5,10-methylenetetrahydrofolate (5,10-CH_2_-THF). TYMS is an enzyme catalysing the conversion of dUMP to dTMP, which is relating to the production of DNA. ATIC catalyses the conversion of tetrahydrofolate to aminoimidazole carboxamide ribonucleotide (AICAR), which is metabolized to AMP (adenosine monophosphate), ADP (adenosine diphosphate) and then ATP (adenosine triphosphate). As a result, MTX acts during DNA, RNA and ATP synthesis and inhibits the cell growth and prolification [49,52,53].

MTX is a highly tetratogenic drug and the use of MTX is prohibited in pregnant women, those who are breastfeeding and also in women who may have a risk of becoming pregnant. The use of MTX is also to be avoided if patients have liver disease, a blood cell disorder, a bone marrow disorder, and particular attention is needed in MTX use if patients have some other diseases such as kidney disease, lung disease, stomach ulcers, and so on. Although the relationship between the pharmacokinetics, or plasma concentrations, and pharmacodynamics of MTX is still obscure, the relationship between plasma MTX concentrations and toxicity is well recognized. The common toxic effects of MTX are nodulosis, hypersensitivity pneumonitis, central nervous system toxicity, postdosing reactions, gastrointestinal symptoms such as nausea, vomiting, abdominal pain and diarrhea, hepatitis with elevated transaminase levels, hematologic abnormalities, rash, alopecia and osteopathy [49]. It is necessary to rescur MTX-induced toxicity if the plasma MTX concentrations exceed 1 × 10^−7^ molar (0.1 μM) for 48 h or more [54]. To rescue MTX-induced side effects, patients are often treated with folinic acid (leucovorin) having a similar chemical structure with folic acid [41,55,56,57]. To decrease the toxic MTX concentrations in the patients, patients are also treated with hemodialysis to eliminate MTX partially, or with carboxypeptidase-G2 (glucarpidase) together with tymidine [58,59,60]. Carboxypeptidase-G2 is a bacterial enzyme and hydrolyzes circulating MTX into inactive metabolite DAMPA to nontoxic levels rapidly. Undestanding the pharmacokinetics of MTX will be essential to predict plasma concentration-time profiles of MTX under various disease states. In this section, the pharmacokinetics of MTX including absorption (oral bioavailability), distribution, metabolism and excretion is reviewed.

### 3.1. Oral Bioavailability of MTX

MTX is used to treat autoimmune diseases such as psoriasis, rheumatoid arthritis (RA) and Crohn’s disease and certain types of cancer such as breast, skin, head, neck, lung, lymphoma, osteosarcoma, and acute leukaemia at markedly different doses. MTX is administered orally, intravenously, subcutaneously and/or intra-muscullarly. In clinical practice, MTX is used at a dosage range from ‘mg’ to ‘g’ levels for the treatments of autoimmune diseases and cancers. In the treatment of autoimmune diseases such as RA and Crohn’s disease, MTX is commonly administered orally at ‘mg’level weekly. The usual starting dose of MTX in RA treatment is 7.5–10 mg per week, and the dose is increased by 2.5–5 mg per week each month to 20–25 mg per week depending on disease activity before considering the treatment a failure, though such escalation of MTX dose may not always result in increased efficacy [61]. The common dosing schedule of MTX is: single weekly oral or intra-muscular low dose; doses divided into two or three weekly doses at consecutive 12h intervals; the dosage may be gradually increased if significant improvement is not noted [53]. Recommendations for use of MTX in patients with RA are published in some countries [62,63,64,65,66].

It is well recognized that MTX exhibits a marked interindividual variability and saturation of the oral bioavailability with increase in the dose of MTX in RA treatments. Henderson *et al.* [67] reported that the absorption of ^3^H-labeled MTX at a dose of 0.1 mg/kg was comparable between oral and intravenous administrations in 10 patients with inoperable solid tumors. Plasma levels following intravenous or small oral doses were directly dose-related and from 54% to 88% of MTX doses was excreted in the urine during the 1st 24 h. At larger doses of MTX, however, gastrointestinal absorption was incompleted, and 4 to 24% of dosed MTX was recovered in urine and stool. Wan *et al.* [68] reported that absorption of 3H-labeled MTX from solution was complete at a dose of 30 mg/m2 body surface area in patients with various types of cancers. At the higher dose (80 mg MTX/m^2^), only 31% of the dose was absorbed in the one patient studied. Balis *et al.* [69] studied the absorption and disposition kinetics of orally administered MTX in children with acute lymphoblastic leukemia (ALL) in a dosing range from 6.3 to 28.1 mg/m^2^.In that study, the fraction of the dose absorbed was scattered in a range from 23% to 95% of dose among 15 children. In addition, the time courses of serum MTX levels in 5 patients at different dosage levels showed significant interindividual variability. At the higher dosage level, absorption of MTX was more prolonged and the absorption percentage of the total dose became smaller [69]. Seideman *et al.* [50] studied the pharmacokinetics of MTX and its hydroxy metabolite in patients with RA. The AUC0-170h of MTX did not differ between intra-muscular and oral administrations, and the excretion of MTX in urine as an intact drug was over 80% when MTX was administered at a dose of 15 mg, and about 3% was excreted as 7-OH-MTX into urine during 24 h after drug administration. These results indicate that MTX is absorbed almost completely after oral administration at a dose of 15 mg. Lebbe *et al.* [70] reported that the interindividual variability of the AUCs of plasma MTX showed a more than five fold, where the intraindividual variability was less than 30% in nine of 10 patients, when MTX was administered orally at a dose of 15 mg in RA patients. Kurnik *et al.* [71] reported that the oral bioavailability of MTX in patients with stable Crohn’s disease was highly variable, and averages 73% of that of subcutaneous administration, when MTX was administered at doses of 15–25 mg. In the study, the concomitant folic acid (5 mg orally or subcutaneously) exerted no significant effect on the bioavailability of MTX. Hoekstra *et al.* [72] reported that the bioavailability of a higher oral dose of MTX (> or = 25 mg weekly) was highly variable in adult patients as follows: Mean bioavailability was 0.64 (range from 0.21 to 0.96) compared to subcutaneous administration, and reported that a change to parenteral administration should be considered to improve efficacy of MTX at dosages of 25 mg weekly or more. Moshkowitz *et al.* [73] reported that orally administered MTX (12.5 mg) was well absorbed in patients with Crohn’s disease, ulcerative colitis or RA, and concluded that MTX was proven to be effective in inflammatory bowel disease after oral administration. Balis *et al.* [74] reported the high variability of plasma MTX concentrations and the considerable interpatient variability in children with lower risk ALL, in which the AUC of MTX ranged from 0.63 to 12 μmol·h/L among children. They also reported that drug dose, patient age, and duration of therapy did not account for the variability, but AUC of MTX was significantly higher in girls than boys (*p* = 0.007). The correlation coefficient (r) for the relationship between the dose and AUC of MTX in ALL children was 0.23, indicating the marked interindividual variability of oral bioavailability of MTX [74]. Herfarth *et al.* [75] reported that pharmacokinetic studies employing MTX suggested that in the dosage range of 15-25 mg an oral application resulted in a significantly lower bioavailability of the drug, at least in a subgroup of patients. The divided doses of oral high-dose MTX is reported to be a rational alternative to intravenous or subcutaneous high-dose MTX [72,75,76,77]. These phenomena also indicate that the intestinal absorption of MTX is saturated at a higher oral dose, and therefore exhibits a marked interindividual variability in the oral bioavailability. The marked interindividual variability of oral bioavailability of MTX may primarily come from the difference in the functional expression levels of specific carrier system, since the intraindividual variability is quite small.

### 3.2. Contribution of Multiple Transporters to Intestinal Absorption of MTX

The mechanism underlying the variability and saturation of oral bioavailability in clinical practice is not yet well understood. The scattering of oral bioavailability of MTX was not ascribed to the food intake or renal failure such as the low GFR [78,79]. In contrast, the increase in the oral bioavailability by the splitting of a higher oral dose of MTX suggests the contribution of saturated transport in oral bioavailability. MTX is known as a substrate of multiple transporters including solute carrier (SLC) influx transporters and ATP-binding cassette (ABC) efflux transporters. Regarding SLC transporters, MTX is transported as a substrate by reduced folate carrier (RFC), proton-coupled folate transporter (PCFT), organic anion transporter 3 (OAT3) and organic-anion transporting polypeptide 1A2 (OATP1A2) [80,81,82,83,84,85]. PCFT and RFC are expressed in the intestine, and PCFT is highly expressed on the brush-boder membranes of the proximal small intestine in rats [86]. The expression site of RFC is not yet determined; some researchers considered that RFC presents primarily on the basolateral domain [87,88,89], some researchers found RFC expressed on the intestinal brush-border membrane in mouse and rat [90,91], and some researchers indicated that RFC are expressed both brush-boder and basolateral membranes. RFC1-mediated transport has a neutral pH optimum and specificity for reduced folate [92,93,94]. In human colonic basolateral membrane, RFC is expressed, and the carrier-mediated folate transport is pH-dependent, DIDS-sensitive, electroneutral and inhibited by MTX [95,96]. PCFT transports both oxidized and reduced folate by using H+ gradient as a driving force [84,97,98]. With Western blot analysis for PCFT, the band density of PCFT in proximal intestine was 4.3-fold stronger than that in distal small intestine of rats [99]. In mice, the expression of mRNA of PCFT, as well as RFC1, in duodenum is also approximately 4-5 fold higher than that in the jejunum [100]. In brush-border membrane vesicles and everted sacs of rat intestine, MTX was transported in saturable and pH-dependent manner [84,101,102], in which the transport of MTX was greatest at pH 4.5 and comparably high in a range from pH 4.0 to 5.5. Above pH 5.5, the transport decreased with an increase in pH and reached a negligibly small level at a neutral pH and above, indicating that PCFT-mediated transport is highly sensitive to extracellular pH [98].

In addition to above SLC transporters, MTX is also transported by ABC efflux transporters such as breast cancer resistance protein (BCRP) and multidrug resistance proteins (MRPs) [103,104,105,106,107]. BCRP is expressed on the apical brush-border membrane of enterocytes preferentially in the distal intestine [108,109], and suppresses MTX absorption in the small intestine. MRP1 ~ MRP6 are expressed in the small and large intestine of man and rodents [110,111,112,113]. In particular, MRP2 and MRP3 have greater roles than other MRPs, because of their higher expression levels in the intestine. MRP2, localized in the brush-border membrane, is preferentially expressed at the proximal intestine, and MRP3, localized in the basolateral membrane, is at the distal intestine [111,114,115,116].

#### 3.2.1. Site-Specific Contribution of PCFT in Intestinal MTX Absorption

The site-specific intestinal absorption of MTX was studied in rat intestine [99], by considering the contribution of SLC transporters such as PCFT and RFC. At pH 5.5, the mucosal influx rate of MTX in proximal intestine was significantly higher than that at pH 7.4. Also, proximal intestine showed greater MTX influx rate than distal intestine. Coadministration of folate or its analogues, such as folinate and 5-methyltetrahydrofolate being substrates for both PCFT and RFC, significantly suppressed the MTX influx at pH 5.5, whereas thiamine pyrophosphate, an inhibitor of RFC1 alone, exerted no significant effect. Benzbromarone, an MRP inhibitor, increased the intestinal absorption of MTX by approximately 1.3 fold at pH 5.5. These results indicated that the intestinal absorption of MTX is mostly mediated by PCFT at the proximal intestine, because PCFT is abundantly expressed at the proximal intestine compared to that at the distal intestine, and MRP can decrease the intestinal absorption of MTX [117]. The gastrointestinal pH in normal human subjects is reported to be pH 1.0–2.5 in the stomach, pH 6.0–6.5 in the proximal small intestine and pH 7.4–7.5 in the mid and distal small intestine in normal subjects [118]. Thus, the variation in luminal pH, especially the higher luminal pH at the proximal intestine, and the decrease in the expression levels of PCFT would cause variation markedly in oral bioavailability among patients. In addition, it was demonstrated that the variation in the gastric emptying rates (GER) can modify the intestinal absorption of MTX, since the absorption window for MTX, or expression site of PCFT, is restricted, as well as the case of riboflavin [119].

#### 3.2.2. Site-Specific Contribution of ABC transporters in Intestinal MTX Absorption

The contribution of ABC transporters such as MRP2, MRP3 and BCRP in intestinal MTX absorption was examined at pH 7.4, at which pH the contribution of PCFT can be negligible, since PCFT works only under acidic condition [99,117]. In rats, the band density of MRP2, located on apical membrane, was approximately 2.8-fold stronger in the jejunum than that in the ileum, and that of MRP3, located on the basolateral membrane, in the ileum was stronger by approximately 1.6-fold of the jejunum. The expression of BCRP was comparable between the jejunum and ileum, though it is generally reported that BCRP is preferentially expressed in the distal intestine. In studies using rat everted intestine, the serosal-to-mucosal efflux rate of MTX at pH 7.4 in the jejunum was greater than that in the ileum. The addition of verapamil, an inhibitor of P-glycoprotein, exerted no significant effect on MTX serosal-to-mucosal efflux in both the jejunum and ileum, indicating that MTX is not transported by P-glycoprotein. In the jejunum, pantoprazole, a BCRP inhibitor, and probenecid, an MRP inhibitor, decreased the mucosal efflux of MTX significantly, indicating that the intestinal absorption of MTX is suppressed by both BCRP and MRP in the jejunum. In contrast, in the ileum, the serosal-to-mucosal MTX efflux was inhibited by pantoprazole alone, indicating that the BCRP suppresses the intestinal absorption of MTX in the ileum. In the efflux study of MTX from the everted intestine in the absence or presence of probenecid [117], MTX was preferentially effluxed from the mucosal side and the effluxed amount was several-fold higher than that from the serosal side in the jejunum. Probenecid significantly suppressed the mucosal MTX efflux by approximately 30% of control, suggesting that the intestinal absorption of MTX can be suppressed by apical MRP2 in the jejunum. In the ileum, the mucosal MTX efflux was lower, and serosal MTX efflux was slightly higher than that in the jejunum, suggesting that there is MRP3-mediated efflux of MTX in the basolateral membrane of the ileum. Collectively, it was demonstrated that the intestinal absorption of MTX is mainly mediated by PCFT in the proximal intestine and inhibited by apical MRP2 and BCRP partly but significantly. In the distal intestine, the intestinal absorption of MTX is facilitated by basolateral MRP3, and partly inhibited by apical BCRP, though the absorption rate of MTX is lower than that in the proximal intestine. The site specific MRPs-mediated bidirectional efflux was also observed with DNP-SG, a typical substrate of MRPs [107,116]. The role of MRP3 in facilitating MTX absorption has also been demonstrated by using Abcc3^-/-^ and wild-type mice [100].

#### 3.2.3. Effect of Gastric Emptying Rates on MTX Oral Absorption in Rats

It is known that riboflavin has a narrow absorption window in the upper part of the small intestine, and therefore, administration after meal or gastro-retentive formulation such as floating pellet, which can reduce the gastric emptying rate (GER) of riboflavin, can increase the oral absorption of riboflavin [120,121,122,123,124]. In general, the reduced delivery rate of a substrate drug to the absorption site can decrease the unabsorbed fraction by avoiding the saturation in the transporter-mediated transport. Such site- specific intestinal absorption has also been reported with various ions and nutrients, including thiamine hydrochloride [125] and bile acids [126,127,128]. The expression site of a specific transporter for MTX, or PCFT, is also restricted in the upper part of the small intestine. In addition, the proton coupled transport system is expected to be active mostly in the upper small intestine having a lower luminal pH. Thus it was considered that the intestinal absorption of MTX can be modified by the alteration of GER, as well as the case of riboflavin. In the study [119], rats were treated with metoclopramide to increase GER or with scopolamine buthylbromide to decrease GER, subcutaneously. In untreated control rats, MTX was absorbed rapidly from the intestine and disappeared from circulating plasma in a first order rate constant. The increase in GER significantly decreased the values of peak plasma level (C_max_) and AUC of MTX to approximately half of those in control rats, possibly due to the saturation in PCFT-mediated transport of MTX. In contrast, the decreased GER delayed the T_max_ significantly, and increased AUC of MTX by approximately 1.4-fold of that in control rats. These results suggest that the intestinal absorption of MTX is mainly dependent on PCFT function, and PCFT expression is localized at the proximal intestine. Taken together, the oral bioavailability of MTX is affected by many factors including the doses of MTX, GERs, and expression levels of PCFT. In addition, the function of intestinal PCFT is known to be modulated by many drugs in clinical practice, such as aminopterin, sulfasalazine, pyrimethamine, benzimidazoles, and proton-pump inhibitors such as omeprazole, pantoprazole, and lansoprazole [86,129,130]. The gastric and intestinal pHs would also affect the oral bioavailability of MTX greatly, because of the low solubility of MTX under acidic condition and H^+^-coupled transport system of PCFT.

### 3.3. Systemic Clearance of MTX

In pharmacotherapy with high-dose MTX for certain type of cancers such as lymphoma, osteosarcoma and acute leukaemia, MTX is administered by intravenous infusion at a dosing range from 300 mg/m^2^ to 12 g/m^2^ over 6–24 h. After such parenteral administration of MTX, MTX is cleared from the body primarily by renal excretion (glomerular filtration and secretion), and partly by the metabolism to 7-OH-MTX. However, the time profiles of plasma MTX concentrations after infusion sometimes show a marked interindividual variability. Pratt *et al.* [131] reported that the half-life for plasma MTX elimination was from 120 to 210 min at terminal phase after administration in a dosage range from 100 to 500 mg/kg, where no significant difference was observed in the urinary excretion percentages (average 95%, and ranged from 77 to 110% of administered drug). Wall *et al.* [132] reported that the dosage adjustments were required in 14 out of 24 patients, with doses ranging from 2,854 to 6,700 mg/m^2^ per course (a 24h infusion), to achieve the target steady-state concentration of 65 μM in children with newly diagnosed ALL. They concluded that dosage individualization decreases interpatient variability and avoids potentially toxic MTX exposures in heavily pretreated ALL patients. A small amount of MTX is excreted into saliva and sweat. Schrøder *et al.* [133] reported the variation of the MTX excretion into saliva and sweat after 24 h continuous MTX infusion (0.5–6 g/m^2^) in 14 patients with various malignant diseases. In the study, there were two patients, whose renal MTX excretions were markedly delayed, and in one of them, the salivary MTX elimination was also retarded. The interindividual variability, or saturation, in systemic clearance of MTX is also observed even at a low dose. Egan *et al.* [134] reported that erythrocyte MTX concentration reached a plateau at approximately 6–8 weeks after the commencing subcutaneous MTX therapy in patients with steroid-requiring Crohn’s disease or ulcerative colitis, and there were no statistically significant differences in mean week 6–16 erythrocyte MTX concentrations between patients randomized to 15 or 25 mg/week, respectively. They also reported that the blood levels of MTX did not predict efficacy or toxicity, though clinical status improved in four out of 11 patients after MTX dose escalation from 15 to 25 mg/week. Joerger *et al.* [135] reported that two patients out of 76 patients showed markedly increased drug exposure when the patients received 3 mg MTX as a 3 h infusion. In the case of clinical therapy, patients are treated not only by MTX but also by various drugs, and the physiological conditions of patients such as the blood flow rates, liver function, renal function would be greatly varied among patients. Also, multiple transporters are contributed in various pharmacokinetic processes of MTX such as the distribution and elimination. It is not easy to study the detailed mechanism of such the saturation and interindividual variability of MTX pharmacokinetics in human patients. 

Animal studies can give clues in understanding the effects of various influencing factors on pharmacokinetics of MTX, though a marked interspecies difference exists in the pharmacokinetics of MTX. For example, different from the case in humans, MTX is preferentially excreted into the bile in rats. Masuda *et al.* [103] reported that the total body clearance (CL_total_) and the biliary clearance (CL_bile_) of MTX after intravenous administration at a dose of 22 μmol/kg were 16.8 mL/min/kg and 12.2 mL/min/kg, respectively, indicating that the biliary excretion of MTX accounts for 73% of total MTX clearance in Sprague Dawley (SD) rats. In Eisai hyperbilirubinemic rats (EHBR) whose MRP2 is defective as a consequence of heredity, the CL_total_ decreased to 7.07 mL/min/kg, and the mean residence time (MRT) of MTX in plasma increased to 76.6 min from 29.5 min. The CL_bile_ decreased to 0.28 mL/min/kg in EHBR, due to the loss of MRP2-mediated biliary excretion for MTX. Similarly, Yokooji *et al.* [136] reported that CL_total_, CL_bile_ and CL_urine_ of MTX were 12.2, 6.6 and 4.9 mL/min/kg, respectively, under a steady-state plasma MTX concentration (24.4 μM), and the values of CL_bile_ and CL_urine_ of MTX were significantly decreased when SD rats were treated with probenecid and bilirubin, in which probenecid and bilirubin (or its glucuronide conjugates) are potent MRP2 inhibitors. These results suggest the contribution of MRP2-mediate biliary and renal excretion of MTX in rats. The uptake of MTX into the liver is mainly mediated by OATP1B1. van de Steeg *et al.* [137] generated a transgenic mouse model with specific and functional expression of human OATP1B1 in the liver. The amount of MTX in the liver increased by approximately 2-fold in the OATP1B1 transgenic mice compared with wild-type mice. They also examined the role of mice OATP1a/1b using mice lacking all Oatp1a/1b transporters and wild-type mice, and demonstrated the pronounced role in determining plasma levels and tissue distribution of MTX [137]. Bremnes *et al.* [138] studied the pharmacokinetics of MTX and 7-OH-MTX in bile, urine, and serum in rats, after short-time infusion of 10, 50, 250, and 1000 mg/kg MTX. In serum, doses exceeding 10 and 50 mg/kg MTX led to a dose-dependent decline in CL_total_ and CL_bile_ as follows: the mean values of CL_total_ were 13.6, 10.1, 6.9, and 5.0 mL/min kg at 10, 50, 250, and 1000 mg/kg MTX, respectively, and those of CL_bile_ were 9.9, 7.5, 3.4, and 1.9 mL/min kg at 10, 50, 250, and 1000 mg/kg MTX, respectively. In contrast, the hepatic metabolism of MTX to 7-OH-MTX was not saturated at the doses examined. In the study, the central volume of distribution, Vc, and the apparent volume of distribution in the postdistribution phase, Vβ, at a dose of 1000 mg/kg MTX were significantly greater than that at a dose of 250 mg/kg MTX, in which the hematocrit values were reduced by 26.1% (average) during the experiments in rats treated at a dose of 1000 mg/kg MTX, whereas a somewhat smaller reduction (mean 21.2%) in hematocrit was observed in the other MTX-treated rats. Moriyasu *et al.* [139] studied the strain difference using four-different strain rats in the pharmacokinetics of 7-OH-MTX and reported that excretion of 7-OH-MTX in bile, feces and urine was highest in Sea:SD rats (6.2%, 4.2%, and 0.8% of dose, respectively) and lowest in WKA/Sea rats (0.02%, 0.2%, and 0.003%, respectively). The variation of excreted amounts of 7-OH-MTX were closely correlated with the strain differences of cytosolic MTX 7-hydroxylase and benzaldehyde oxidase activities and the variation of 7-OH-MTX formation from MTX *in vivo* in rats was due primarily to variation of aldehyde oxidase activities.

In human, the systemic clearance of MTX is mostly due to the renal clearance. In the renal uptake process of MTX from circulating blood, MTX is considered to be mediated by RFC1 and human organic anion transporters such as OAT1 and OAT3, localized at the basolateral membrane of the proximal tubule [87,88,140,141,142]. At the apical membrane of the proximal tubule, MTX could be eliminated into the renal tubule by MRP2 and BCRP or taken up by OAT4, organic-anion transporting polypeptide transporter (OATP) 1A2 [82], or folate receptors [143]. PCFT, localized on brush border membrane of polarized epithelia, may also contribute in the recovery of MTX from renal tubular fluid [87,88]. MTX has been used in combination with nonsteroidal anti-inflammatory drugs (NSAIDs) in the treatment of inflammatory diseases and malignancies, and their drug-drug interactions in the renal process are extensively investigated by many research groups. Tracy *et al.* [144] reported the effect of NSAIDs on the pharmacokinetics of MTX after oral administration in 10 patients with RA, in which the oral and renal clearances of MTX were 11.0 L/h and 7.9 L/h, respectively. The percentage of MTX excreted unchanged was 72% of the dose and plasma unbound fraction (fu) was 0.54. These parameters of MTX including oral clearance, renal clearance, percentage excreted unchanged and fu varied no more than 12.2% from the non-NSAID control during concomitant administration of ketoprofen (3 mg/kg/day, for >6 days), flurbiprofen (3 mg/kg/day, for for >6 days), or piroxicam (20 mg/day, for >13 days), and were not statistically different from non-NSAID control. Takeda *et al.* [142] reported that MTX is taken up via OAT3 and OAT1 at the basolateral side of the proximal tubule and effluxed or taken up at the apical side via OAT4, and OAT1, OAT3, and OAT4 are the sites of drug interactions between MTX and NSAIDs, probenecid and penicillin G in human. Nozaki *et al.* [81] examined the drug-drug interactions using rat kidney slices and reported that rat OAT3 and RFC1 are almost equally involved in the uptake of MTX by the kidney slices. NSAIDs, except salicylates, were potent inhibitors of rat OAT3, but weak inhibitors of RFC1, suggesting that the renal uptake of MTX is not so greatly affected by NSAIDs as expected from the inhibition of rat OATs-mediated transport. El-Sheikh *et al.* [145] reported that salicylate, piroxicam, ibuprofen, naproxen, sulindac, tolmetin and etodalac inhibited MRP2- and MRP4-mediated MTX transport, diclofenac inhibited MRP4-mediated transport, indometacin and ketoprofen inhibited MRP2-mediated transport, and phenylbutazone stimulated MRP2-mediated transport. VanWert and Sweet [85] studied the role of mouse OAT3 in the disposition of MTX, using knockout mice, and concluded that OAT3 contributes to MTX clearance, but represents only one component responsible for the elimination of MTX. The effect of MTX treatment on the expression of above transporters is also reported. Shibayama *et al.* [83] examined the effect of single injection of MTX intraperitoneally on the expression of MRP2, BCRP, and OATs in rats, and reported the decreases of MRP2 levels in the liver and ileum, renal OAT1 and OAT3, mRNA levels of constitutive androstane receptor (CAR) and pregnane X receptor (PXR), four days after injection of MTX (150 mg/kg) and the recovery of MTX effect by leucovorin. Shibayama *et al.* [146] examined the effect of MTX treatment (150 mg/kg) on the expression of OATP2, P-glycoprotein and bile salt export pump (BSEP) in rats, and reported the down regulation of hepatic OATP2, P-glycoprotein, BSEP, and kidney and ileum P-glycoprotein four days after MTX treatment and partial recovery of MTX effect by leucovorin.

### 3.4. Tissue Distribution of MTX

Three folate transporters including RFC, PCFT and folate receptor-α account for most, if not all, folate influx activities observed in mammalian cells. RFC and PCFT are ubiquitously expressed in normal and malignant mammalian tissues [147]. There are three human folate receptors (α, β, γ) that transport substrates by receptor-mediated endocytosis, and the folate receptor α is ubiquitously expressed and participates in the cellular uptake of folate [148]. Spinella *et al.* [149] compared the transport of MTX between folate receptor-α-mediated transport and RFC-mediated transport in L1210 leukemia cells, and reported that folate receptor-α expressed at sufficient levels can mediate influx of MTX and folates into cells at rates comparable to the RFC. Several articles reported that the affinity of MTX to folate receptor is much low than that to RFC [150]. Deng *et al.* [151] synthetized a series of antifolate inhibitors of purine biosynthesis as selective folate receptor α and β substrates and as antitumor agents, so that compounds have high affinity for folate receptors over RFC and PCFT for entry to tumor cells. Taken together, RFC is thought to play an essential role in physiological folate homeostasis and cancer chemotherapy with MTX at a physiological pH [152]. Hosoya *et al.* [153] reported that although both RFC1 and PCFT mRNA are expressed in isolated rat retinal vascular endothelial cells, the expression level of RFC1 mRNA was 49-fold greater than that of PCFT. In rats, high levels of RFC1 transcript were detected in colon, kidney, brain, thymus and spleen. Moderate rat RFC1 gene expression was observed in small intestine, liver, bone marrow, lung and testes, whereas transcript levels were negligible in heart, skeletal muscle or leukocytes. The expression level of rat RFC1 protein was high in the apical membrane of tunica mucosa epithelial cells of small intestine and colon, the brush-border membrane of choroids plexus epithelial cells, endothelial cells of small vessels in brain and heart, basolateral membrane of renal tubular epithelial cells, plasma membrane of periportal hepatocytes, and in sertoli cells of the testis [152].

### 3.5. Formation of MTX Polyglutamate in Cells

MTX and 7-OH-MTX taken up by cells is metabolized to MTX polyglutamates (MTX-PGs) by folylpoly-γ-glutamate synthetase in various cells, including tumor samples obtained from patients and normal human cells [154,155]. For example, normal myeloid precursor cells converted MTX to MTX-PGs in a concentration- and time-dependent manner, preferentially retaining MTX-PGs with three to seven glutamyl moieties. The formation of MTX-PGs in tumor cells is thought to be important determinant of the cytotoxicity in *in vitro* and *in vivo* systems by many investigators. Fabre *et al.* [156] assessed MTX cytotoxicity by clonogenic assay in agar with granulocytic progenitor cells from mouse bone marrow and in the Ehrlich ascites tumor, the K562 human chronic myelogenous leulemia, and the P388 murine leulemia. After a 2 h exposure to MYX, the concentrations necessary to produce 50% inhibition of colony formation were 100, 25, 1.2, and 0.25 μM, respectively, which was inversely related to the ability of the tumor cells to accumulate MTX-PGs, suggesting the marked differences in the accumulation of MTX-PGs between the tumor cells. Jolivet and Chabner [157] measured the dissociation half lives of MTX (Glu1), MTX-Glu2, MTX-Glu3, MTX-Glu4 and MTX-Glu5 in the binding to dihydrofolate reductase (DHFR) in ZR-75-B human breast cancer cells, and reported that their half lives were 12, 39, 102, 108 and 120 min, respectively. These results suggested that the longer chain polyglutamates have prolonged intracellular retention and can be dissociated less readily than MTX-Glu2 from DHFR. Fabre and Goldman [45] compared the cytotoxicity of 7-OH-MTX and MTX. After 2 h exposure, the 50% inhibitory concentrations for 7OH-MTX and MTX in cells growing agar were 10-5 and 10-6 M, respectively, indicating a 10-fold difference in cytotoxicity between them, and reported that, as observed for MTX, the 7-OH-MTX-PGs are retained within the cells and have a sustained cytotoxic effect after the monoglutamate is removed. Synoid *et al.* [158] determined the concentrations of MTX and MTX-PGs in bone marrow blasts obtained from 101 children randomized to single-agent therapy with either high-dose MTX or low-dose MTX. Blast concentrations of total MTX-PGs and of long-chain MTX-Glu4–MTX-Glu6 were both significantly higher after high-dose MTX, and MTX-PGs were significantly higher in B-lineage blasts than in T-lineage blasts. Masson *et al.* [159] examined the relationship between MTX-PGs concentrations and its antileukemic effects in 150 children with newly diagonosed ALL treated with either high-dose MTX or low-dose MTX, and reported that the extent of inhibition of de novo purine synthesis in ALL blasts was significantly related to the blast concentrations of long-chain MTX-PGs. These data indicate that higher blast concentrations of MTX-PGs are associated with greater antileukemic effects. Dalrymple *et al.* [160] determined MTX-Glu1–MTX-Glu5 concentrations in red blood cells of patients with RA, and reported that the median times to reach steady state in red blood cells (defined as 90% of the maximum concentration) were 6.2, 10.6, 41.2, 149, and 139.8 weeks, respectively, and median half-lives of elimination were 1.2, 2.3, 4.3, 2.7 and 2.1 weeks for MTX-Glu1 (or MTX), MTX-Glu2, MTX-Glu3, MTX-Glu4, and MTX-Glu5, respectively. They also reported wide interpatient variability of the accumulation and elimination of these MTX-PGs in red blood cells of adult patients with RA. Stamp *et al.* [161] examined nongenetic factors that influence MTX-PG concentrations in red blood cell of patients receiving long-term stable low-dose oral MTX. They reported that increased age, lower estimated glomerular filtration rate (GFR), MTX dosage, longer duration of MTX treatment, and use of prednisone were associated with significantly higher MTX-PGs concentrations. Smoking and folate levels also affect MTX-PGs concentrations in red blood cells. They also tried to define a therapeutic range of red blood cell MTX-PGs concentrations, including threshold values for efficacy and adverse effects in patients receiving long-term oral MTX treatment in patients with RA, and reported that there was no relationship between MTX-PGs concentrations and adverse effects [162]. Dervieux *et al.* [163] measured red blood cell MTX-PGs concentrations in adult RA patients, and reported that the selective emergence of long-chain MTX-PGs is function of dose, time of exposure and hence dosage intensity, and concluded that switching from oral to parenteral MTX produces a selective accumulation of longer chain MTX-PGs that are known to be more potent inhibitors of de novo purine biosynthesis than shorter chain MTX-PGs. Though the response to and toxicity of MTX are unpredictable in patients with juvenile idiopathic arthritis, intracellular MTX-PGs concentrations have been demonstrated to be a promising predictor of drug response [159]. Becker *et al.* [164] measured red blood cell MTX-PGs concentrations to determine the predictors of MTX-PGs variability in patients with juvenile idiopathic arthritis, in which concentrations of MTX-PGs (MTX-Glu1–MTX-Glu7) were determined. They reported that the route of MTX administration was significantly associated with MTX-Glu1~ MTX-Glu5 subtypes: higher concentrations of sum of MTX and MTX-Glu2 were observed in patients receiving oral doses of MTX, whereas higher concentrations of sum of MTX-Glu3, MTX-Glu4 and MTX-Glu5 were observed in patients receiving subcutaneous doses of MTX. Then they investigated the genetic predictors of MTX-PGs variability and associations between MTX-PG and drug response in patients with juvenile idiopathic arthritis, in which genotyping for 34 single-nucleotide polymorphisms (SNPs) in 18 genes within the MTX metabolic pathway was performed [165]. They reported that the cluster with high concentrations of sum of MTX-Glu3 ~MTX-Glu5 was associated with elevated liver enzyme levels on liver function tests, and there were higher concentrations of sum of MTX-Glu3 ~MTX-Glu5 in children who reported gastrointestinal side effects and had abnormal findings on liver function tests. No association was noted between MTX-PGs and active arthritis.

As described already, members of MRP family, notably MRP1-5 and BCRP have been recognized as cellular exporters for MTX [166,167,168,169,170,171]. MTX-PGs are also transported by multiple ABC efflux transporters. Chen *et al.* [172] reported that BCRP (ABCG2) transports MTX-Glu2 and MTX-Glu3, but not MTX-Glu4, in membrane vesicles prepared from HEK293 cells transfected with ABCG2 expression vector. Similar finding is also reported by using stably transfected cell lines expressing either wild-type (Arg482) or mutant (Gly482) variants of BCRP by Volk and Schneider [173]. Rhee and Schneider [174] examined the effect of BCRP on MTX-PGs accumulation in intact cells, and concluded that BCRP overexpression can cause a reduction in total MTX accumulation as well as the reduction in the proportion of long-chain MTX-PGs. The concentration-dependence of MTX-Glu2 uptake by BCRP is also reported by Breedveld *et al.* [105]. Wielinga *et al.* [169] reported that MTX-Glu2, but not MTX-Glu3, was transported by MRP5, and that MTX-Glu2 level was strongly reduced in MRP5-overexpressing HEK293/MRP5I cells, indicating the contribution of MRP5 to resistance against antifolate drugs.

### 3.6. Effect of Polymorphisms on Methotrexate Pharmacokinetics

In the pharmacokinetics and pharmacodynamics of MTX, multiple transporters and metabolic enzymes are involved. Thus, the polymophisms of the gene of such transporters and/or enzymes may modify the pharmacokinetics and pharmacodynamics greatly. For example, polymorphisms in the genes of folate metabolism enzymes are known to be associated with some forms of cancer such as colorectal neoplasia, adult ALL and non-Hodgkin’s lymphoma (NHL) [175,176]. De Jonge [177] studied whether common polymorphisms in genes involved in folate metabolism affect MTX sensitivity, and found that polymorphisms in the folate-related genes methylenetetrahydrofolate reductase (MTHFR 677C > T, MTHFR 1298A > C), methionine synthase reductase (MTRR 66A > G), and serine hydroxymethyl transferase (SHMT1 1420 C > T) are related to MTX resistance in pediatric patients with ALL. The polymorphisms of OATP1B1 gene generally decrease the transporting activity of OATP1B1, which is expressed on the sinusoidal membrane of human hepatocytes and mediates the hepatic uptake of many endogenous and xenobiotic substances including MTX [178]. Treviño *et al.* [179] examined the gene basis of interindividual variation in MTX pharmacokinetics in children with ALL, and found that two SNPs in OATP1B1 gene were associated with GI toxicity of MTX. Recently Lopez-Lopez *et al.* [180] analyzed 10 polymorphisms in seven genes [MTHFR, TS, SHMT1, RFC1, ABCB1, ABCG2, and SLCO1B1 (OATB1B1 gene)] from the MTX metabolism in Spanish pediatric B-ALL patients, and found a statistically significant association between MTX plasma concentration and the SLCo1B1 rs11045879 CC genotype or rs4149081 AA genotype. They did not find any significant association in the other genetic polymorphisms analyzed. Ramsey *et al.* [181] examined the effects of rare (a minor allele frequency of <1%) and common (>50%) variants in SLC1B1 on MTX disposition, and found that common non-synonymous (NS) variants accounted for the majority, but rare damaging NS variants constituted 17.8% of SLCO1B1’s effects (1.9% of total variation) had larger effect sizes than common NS variants.

### 3.7. Summary for Methotrexate Pharmacokinetics

Various SCL and ABC transporters are contributed in the pharmacokinetics of MTX. A marked interindividual variability of MTX pharmacokinetics may come from such complicated biomembrane transport systems in absorption, distribution and renal elimination processes of MTX, though the contribution of metabolism process is considered to be relatively small. In particular, the interindividual variability of intestinal absorption of MTX is quite large, due to the restricted absorption window for MTX, H^+^-coupled transport system of PCFT, and low solubility of MTX under acidic condition. On one hand, it is also reported that the intraindividual variability of MTX pharmacokinetics including oral absorption is relatively small. Appropriate TDM of serum MTX [182,183] and rescue treatment with leucovorin for MTX toxicity will gain reliable and effective MTX treatment, though careful attention for the possible occurrence of various side effects is necessary.

## 4. Conclusions

The combination therapy with mizoribine and methotrexate is known to be effective to control the symptoms of rheumatoid arthritis in patients with an insufficient clinical response to methotrexate alone. As reviewed in this article, however, both drugs may show a significant interindividual, but not intraindividual, variability in their oral bioavailability, mostly due to the polymophisms of transporter genes. In such case, TDM work of both mizoribine and methotrexate is expected to improve their clinical efficacy in the treatment of rheumatoid arthritis.
